# Expressions and clinical significances of CD133 protein and CD133 mRNA in primary lesion of gastric adenocacinoma

**DOI:** 10.1186/1756-9966-29-141

**Published:** 2010-11-07

**Authors:** Ji-wei Yu, Peng Zhang, Ju-gang Wu, Sheng-hua Wu, Xiao-qiang Li, Shi-ting Wang, Rui-qi Lu, Xiao-chun Ni, Bo-jian Jiang

**Affiliations:** 1 Department of General Surgery, No. 3 People's Hospital, Shanghai Jiao-tong University School of Medicine, Shanghai 201900, China; 2 Department of General Surgery, Henan Province People's Hospital, Zhengzhou, Henan 450000, China; 3 Department of Pathology, No. 3 People's Hospital, Shanghai Jiao-tong University School of Medicine, Shanghai 201900, China; 4 Experimental Center, No. 3 People's Hospital, Shanghai Jiao-tong University School of Medicine, Shanghai 201900, China

## Abstract

**Background:**

To study on expressions and clinical significances of CD133 protein and CD133 mRNA in primary lesion of gastric adenocarcinoma (GC).

**Methods:**

Expressions of CD133 protein by immunostaining (99 cases) and CD133 mRNA by semi-quantitative RT-PCR (31 cases) were detected in primary lesion and in noncancerous gastric mucosa tissue (NCGT). Correlations of CD133 protein expression with clinicopathological parameters and post-operative survival were analyzed. Relations of CD133 mRNA level with Ki-67 labeling index (LI), and lymphatic metastasis were assessed too.

**Results:**

Brown particles indicating CD133 protein positivity occurred in some parts of tumor cells and epithelium. Expressive percentage of CD133 protein positivity was significantly higher in subgroups with >5 cm diameter (*P *= 0.041), later TNM stage (*P *= 0.044), severer lymph node metastasis (*P *= 0.017), occurrences of lymphatic invasion (*P *= 0.000) and vascular invasion (*P *= 0.000) respectively. Severer invasion depth (*P *= 0.011), lymph node metastasis occurrence (*P *= 0.043) and later TNM stage (*P *= 0.049) were the independent risk factors for CD133 protein expression. Average brightness scale value (BSV) of CD133 mRNA was significantly higher in subgroups with >5 cm diameter (*P *= 0.041), lymph node metastasis occurrence (*P *= 0.004) and in lower Ki-67 LI (*P *= 0.02). Relative analysis revealed that BSV of CD133 mRNA related positively to metastatic lymphatic nodes ratio (*P *= 0.008) and metastatic lymph node number (*P *= 0.009), but negatively to Ki-67 LI (*P *= 0.009). Survival of positive subgroup of CD 133 protein was significantly poorer (*P *= 0.047). Lymph node metastasis occurrence (*P *= 0.042), later TNM stage (*P *= 0.046) and CD 133 protein positive expression (*P *= 0.046) were respectively the independent risk factors to survival.

**Conclusion:**

Higher expressive level of CD133 mRNA is associated to lower Ki-67 LI and severer lymphatic metastasis. Therefore, the expressive level of CD133 mRNA can play an appropriate role to reflect the status of lymph node metastasis and proliferation of GC. CD133 protein expression is closely related with larger tumor, later TNM stage, lymphtic metastasis and survival of GC.

## Background

Gastric cancer is among the most common form of cancer of the digestive system with an estimated incidence of approximately 22000 cases in the USA for 2008 [1], and is still one of the most common cancer-related causes of death in the world, particularly in Asian countries [2]. Worldwide, gastric carcinoma is the third most common form of cancer with overall 5-year survival rate of less than 20% as most patients are diagnosed late and are unsuitable for curative surgery. With the challenge of disseminated disease at the time of diagnosis, there is a critical need for finding more effective ways to eradicate the cancer cells. However, the process of cancer initiation, metastasis and recurrence is sequential and selective, and consists of a series of independent steps with interlinks [3-6].

Reportedly, CD133 expressing cells in glioblstoma and colorectal cancers include, but are apparently not limited to, the small subpopulation of tumor cells termed as cancer stem cells (CSCs) which mediate tumor initiation, metastasis and recurrence [4-6], and possess the unique self-renewal properties, the multiple differentiating potential, the proliferating aptitude and the carcinogenesis [5,7,8]. In addition to being considered as the tumor initiating cell population, CSCs have also been demonstrated to resistance to chemotherapy and radiotherapy implying that they are responsible for tumor recurrence [9,10]. At the same time, CD133 has been considered as a CSCs marker in many kinds of tumors such as colorectal [5,6], brain [4,7], prostate [8], pancreatic [11] and gastric cancers [12].

One of the aims in this study was to investigate the expression levels of CD133 protein and CD133 mRNA in primary lesion of gastric adenocarcinoma (GC) and to compare these expressive levels with clinicopathological characteristics and survival time after curative resection. Additionally, we explored the relation of CD133 mRNA expression level with lymphatic vessel infiltration, lymph node metastasis and metastatic lymph node ratio [13] which factors reflected the status of lymphatic metastasis demonstrated wildly as one of the main risk factors for the prognosis. At the same time, immunostaining for Ki-67, a kind of cellule nucleus protein, and its labeling index (LI) were applied to assess the proliferating ability of tumor cells with higher or lower CD133 mRNA level and the relation of this proliferating ability of tumor cells sharing higher or lower CD133 mRNA level were evaluated.

## Methods

### Patients

A total of 99 patients who underwent radical gastrectomy (D_2 _or D_3_; R_0 _or R_1_) for primary GC at our hospital from July 2004 to July 2009 were registered for immunohistochemical staining in this study. The median age of the patients was 62.0 years old (range 29~83 years old) in this group of patients. Among them, a total of 31 patients from May 2008 to July 2009 were also assessed by semi-quantitative RT-PCR for detecting CD133 mRNA in primary lesion and in noncancerous gastric tissue (NCGT), which was identified by pathological observation, at > 5 cm distance adjacent to primary lesion, and by immunohistochemical staining for Ki-67 expression in tumor cells. In this group of patients, the median age of the patients was 64.0 years old (range 34~83 years old). None of them accepted any preoperative chemotherapy or radiotherapy. All of the cases received postoperative adjuvant chemotherapy. The diameter of tumors was ranged from 1 to 10 cm; median 5.0 cm. Preoperative informed consent was obtained from each patient included in the study in accordance with institutional guidance. Half specimen from primary lesion or NCGT was fixed in 10% buffered formalin and embedded in paraffin. In this part of sample, full layer of gastric wall was included for next stainings. Three sections from each sample of primary lesion were serially cut for HE staining, CD133 and Ki-67 immunostainings. Another half specimen, mainly from the selected mucosa layer was used for PCR detection, was fixed in fluid nitrogen and then stored in -80°C until use. This study was approved by ethic committee of our hospital before its start.

### Immunohistochemical and pathological examinations

Serial tissue sections with 4 μm were stained for CD133 (CD133/1 monoclonal antibody; 1:40 dilution, Miltenyi Biotec GmbH, Bergisch Gladbach, Germany) by ABC method (mouse ABC Staining System, sc-2017, Santa Cruz Biotechnology Co, CA, USA), Ki-67 (mouse against to human of monoclonal antibody, Changdao Biotech, Co., Shanghai, China) by two steps method [14] and HE section. In details for CD133 immunostaining, sections were dewaxed, and rehydrated by sequential immersion in xylene, graded ethanol, and water. Antigen retrieval was done by heating the slides in microwave oven in 0.01 mmol/L citrate buffer (pH 6.0). After washing in phosphate-buffered saline (PBS), the slides were exposed to 10% normal blocking serum (Santa Cruz Biotechnology, CA, USA) for 10 min to reduce the nonspecific antibody binding Endogenous peroxidase activity was blocked by 3% hydrogen peroxide in methanol for 30 min. Incubation with primary antibody of CD133 (50 ul, 1:40 dilution) was performed for one hour at room temperature. And then, immunodetection was performed by ABC staining system according to the production instructions. Primary antibodies were visualized with DAB solution (Santa Cruz Biotechnology Co, CA, USA). Finally, slides were couterstained with haematoxylin to show the nucleus of cells clearly. Cells with brown color as CD133 protein expression in the gland parietes, the cellular membrane surface and the epithelium were considered as positivity of CD133 immunostaining. Negative controls for CD133 and Ki-67 were carried out as above by substituting normal serum for the primary antibodies. Sections from previously studied cases of GC known to positive expression were used as positive controls. Positive percentage as Ki-67 LI was calculated according to the positive cells number in 1000 counted cells number under × 400 magnifications in 5 fields freely selected under a light microscope [14]. All sections were observed and scored by two independent investigators blind to each patient's status.

### RNA isolation and reverse transcriptase polymerase chain reaction (RT-PCR)

Total RNA was extracted from 80-100 mg frozen GC tissue treated with RNA PCR Kit (TaKaRa Biotechnology, Tokyo, Japan) following the manufacturer suggested protocols. Oligo dT-Adaptor Primer was used with AMV reverse transcriptase XL for cDNA synthesis from 500 ng of total RNA. PCR was conducted with TaKaRa *Ex Taq *HS DNA polymerase in 50 μl reaction volumes. Primers (synthesized by Sangon Technology, Shanghai, China) used were including GAPDH (sense, 5'-ACGGATTTGGTCGTATTGGGCG-3'; antisense, 5'-CTCCTGGAAGATGGTGATGG-3') with a product length of 197 bp and CD133 (sense, 5'-TTACGGCACTCTTCACCT-3'; antisense, 5'-TATTCCACAAGCAGCAAA-3') with a product length of 172 bp. The reactions were conducted for GAPDH as the internal control under the following conditions: initial denaturing step at 95°C for 1 min, 28 cycles of 95°C for 1 min, 55°C for 1 min, 72°C for 1 min, followed by 72°C for 10 min; For CD133: initial denaturing step at 94°C for 2 min, 28 cycles at 94°C for 30 seconds, 51°C for 30 seconds, 72°C for 30 seconds, followed by 72°C for 10 min. according to the manufacturer's instruction Five μl CD133 PCR and 2 μl of the products amplified by MyCycler™ Thermal Cycler (Bio-Red Laboratories, CA, USA) were separated on a 1.5% agarose gel (Gene Tech, Shanghai, China) by electrophoresis apparatus (Tunon, EpS 100, Shanghai Tian-neng Tech Co. Shanghai, China). Digital images to exposure the occurrence of CD133 mRNA as a white target strip were captured on a gel documentation system (UNIVERSAL HOOD II, Bio-Red Laboratories, Segrate, Milan, Italy). Imaging assessments to measure the brightness scale value (BSV) of CD133 automatically from the write strip and to compared the relative ratio between CD133 strip and control strip were carried out by Quantity One 1-D analysis software (The Discoveries™ Quantity One 1-D Analysis Software Version 4.5, Bio-Red Laboratories, CA, USA.).

### Clinicopathological assessments

Clinicopathological parameters included gender, age, tumor size histological grade, invasion depth, lymph node metastasis, TNM stage, lymphatic vessel infiltration, vascular infiltration and metastatic lymph node ratio for CD133 protein and CD133 mRNA assessments respectively [13,15], mainly according to UICC classification [15]. And Ki-67 LI was also used in the evaluation of CD133 mRNA expression.

### Prognostic analysis

The deadline of follow-up for 99 patients was until November 2009, and the average survival time was 26.76 ± 17.02 months. A total of 9 cases (9.1% patients) lost in follow-up period. In this registered group, 39 cases died of the recurrence of gastric cancer, vascular diseases of brain or heart, or complications after surgery respectively. All patients in this group for survival assessment were divided as positive or negative subgroup of CD133 immunostaining.

### Statistics

All statistical analyses were performed with the SPSS software version 13.0 (SPSS, Chicago, IL, USA). The correlations between expression of CD133 protein and clinicopathological parameters were assessed with the chi-squared test as a univariate analysis. Furthermore, the multivariate analysis was carried out with Logistic analysis for these correlations in order to explain the more important significances of the observed parameters. CD133 mRNA data was expressed as means ± SD, and statistical analysis was carried out using Student's *t *test. Relative evaluations of CD133 mRNA level with several clinicopathological data were made by Spearman's rho analysis. The Kaplan-Meier method was used to estimate survival as a function of time, and survival differences were analyzed by Log-rank test. The Cox regression model was used for multivariate analysis of prognostic factors. In all of the tests, a *P *value less than 0.05 was considered to be statistically significant.

## Results

### CD133 protein expression in primary lesion

**Figure 1:**
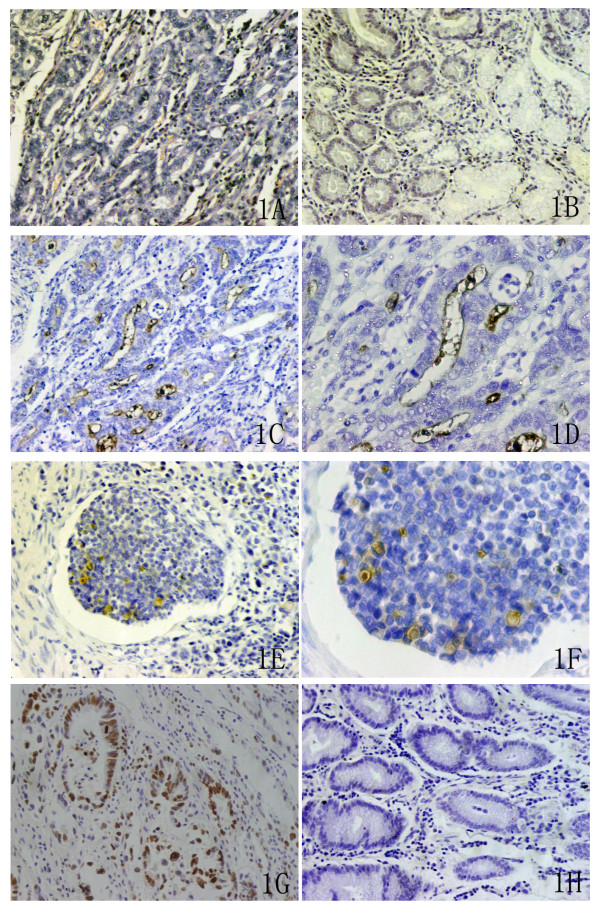
**Morphological observation on the tumor cells with CD133 protein and Ki-67 immunostainings in primary lesion**. Note: A showed HE staining for GC tissue (×200). B showed CD133 immunostaining for NCGT (×200). C (×200) and D (×400) showed CD133 immunostaining for GC tissue. E (×200) and F (×400) showed tumor cells with CD133 positivity in the cancerous emboli in vessel-like structure. G (×200) and H (×200) showed the higher positive and the lower positive expressions of Ki-67 immunostaining (×200) respectively.

Particles sharing brown color indicated to CD133 protein expression occurred in some parts of gland parietes, cellular membrane surface of some tumor cells and some epithelium in primary lesion, in which CD133 positive particles mainly located in some parts of tumor cells in the mucosa and the submucosa layers (Figure 1C and 1D). Some CD133 positive cells were identified in the wall of crypts and in the cancerous emboli in vessel-like structures in primary lesion (Figure 1E and 1F). No positive staining was seen in NCGT as control subgroup (Figure 1B), which positivity rate of CD133 (0%) was significantly lower than that in cancerous subgroup (29.3%, 29 cases/99 cases, *P *= 0.000).

### Correlation of CD133 protein expression with clinicopathological parameters

CD133 expression was significantly correlated with tumor diameter of > 5 cm (*P *= 0.041), severer lymph node metastasis (*P *= 0.017), later TNM stage (*P *= 0.044), occurrences of lymphatic vessel infiltration (*P *= 0.000) and vascular infiltration (*P *= 0.000) (Table 1). Furthermore, with the increase of invasion depth of tumor, the expressive rate of CD133 raised obviously, but no statistical significance. However, further stratified analysis revealed that the expressive rate of CD133 in subgroup of T_3_-T_4 _(6.06%, 6 cases/99 cases) was significantly higher than that in subgroup of T_1_-T_2 _(23.23%, 23 cases/99 cases, *P *= 0.038). The multivariate evaluation by Logistic analysis demonstrated that invasion depth (*P *= 0.011), lymph node metastasis (*P *= 0.043) and TNM stage (*P *= 0.049) were the independent risk factors for CD133 protein expression respectively (Table 2).

**Table 1:** Correlation between CD133 protein expression and clinicopathological features [n(%)](n = 99 cases)

Clinicopathological parameter	Grouping	CD133 protein expression		**χ**^**2 **^**value**	*P *value
				
		positive	negative		
**Gender**	**male**	18(18.18)	51(51.52)	1.130	0.288
	**female**	11(11.11)	19(19.19)		
**Age(year)**	≤ **60**	9(9.09)	30(30.30)	1.200	0.273
	**> 60**	20(20.20)	40(40.40)		
**Tumor diameter(cm)**	≤ **5**	17(17.17)	40(40.40)	4.175	0.041
	**> 5**	12(12.12)	10(10.10)		
**Histological grade**	**1**	5(5.05)	16(16.16)	2.030	0.566
	**2**	13(13.13)	27(27.27)		
	**3**	11(11.11)	27(27.27)		
**Invasion depth**	**T**_**1**_	0(0.00)	11(11.11)	6.116	0.106
	**T**_**2**_	6(6.06)	17(17.17)		
	**T**_**3**_	10(10.10)	21(21.21)		
	**T**_**4**_	13(13.13)	21(21.21)		
**Lymph node metastasis**	**N**_**0**_	3(3.03)	27(27.27)	10.227	0.017
	**N**_**1**_	15(15.15)	20(20.20)		
	**N**_**2**_	7(7.07)	19(19.19)		
	**N**_**3**_	4(4.04)	4(4.04)		
**TNM stage**	II	2(2.02)	19(19.19)	8.108	0.044
	**III**	4(4.04)	10(10.10)		
	**IV**	13(13.13)	31(31.31)		
	**IV**	10(10.10)	10(10.10)		
**Lymphatic vessel infiltration**	**positive**	28(28.28)	27(27.27)	27.636	0.000
	**negative**	1(1.01)	43(43.43)		
**Vascular infiltration**	**positive**	28(28.28)	15(15.15)	46.624	0.000
	**negative**	1(1.01)	55(55.56)		

**Table 2:** Logistic analysis on the correlation of CD 133 protein expression with clinicopathological parameters (n = 99 cases)

Parameter	B	SE	Wald	df	**Sig**.	Exp(B)	95.0%CI for Exp(B)
**Gender**	0.012	0.017	0.201	1	0.328	1.003	0.972~7.873
**Age(year)**	0.007	0.018	0.158	1	0.691	1.007	0.875~3.125
**Tumor diameter(cm)**	0.209	0.123	2.908	1	0.088	1.233	1.334~8.911
**Invasion depth**	-1.238	0.488	6.430	1	0.011	0.290	1.079~12.381
**Histological grade**	0.181	0.281	0.414	1	0.520	1.198	0.987~3.212
**Lymph node metastasis**	-0.929	0.459	4.102	1	0.043	0.395	1.156~18.324
**TNM stage**	1.048	0.636	2.720	1	0.049	2.853	1.138~14.216
**Lymphatic vessel infiltration**	0.847	0.601	1.568	1	0.067	3.213	1.335~10.954
**Vascular infiltration**	0.760	0.662	1.317	1	0.251	2.137	0.991~6.872

### CD133 mRNA expressions in primary lesion and in NCGT

**Figure 2:**
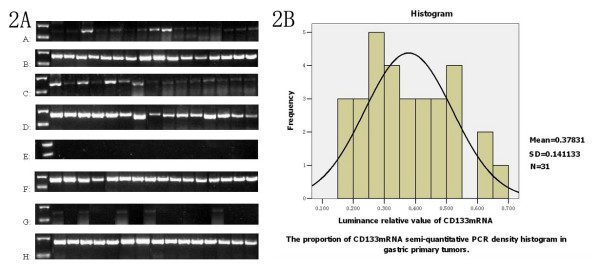
**Detection and distribution of semi-quantitative BSV of CD133 mRNA by RT-PCR (n = 31 cases)**. Note: 2A showed the detection of semi-quantitative BSV of CD133 mRNA. A and C showed CD133 mRNA expressions in primary lesions. E and G showed CD133 mRNA expressions in NCGT. B and D showed GAPDH mRNA expressions as an internal reference for subgroup of primary lesions. F and H showed GAPDH mRNA expressions as an internal reference for subgroup of NCGT. 2B showed the distribution of semi-quantitative BSV of CD133 mRNA.

The semi quantitative RT-PCR detection in 31 patients was performed to confirm the expressions of CD133 mRNA in primary lesion (100.0%) and NCGT (16.1%, 5 cases/31 cases)(χ^2 ^= 15.125, *P *= 0.000) (Figure 2A). Average BSV of CD133 mRNA was 0.3783 ± 0.1411 in primary lesion subgroup and 0.0381 ± 0.0919 in NCGT subgroup respectively (Z = -6.533, *P *= 0.000) (Figure 2B). In comparison with average BSV of CD133 mRNA in NCGT subgroup, the increasing range of average BSV of CD133 mRNA was up to 993% in primary lesion subgroup.

### Correlation of BSV of CD133 mRNA with clinicopathological parameters and Ki-67 LI

**Figure 3:**
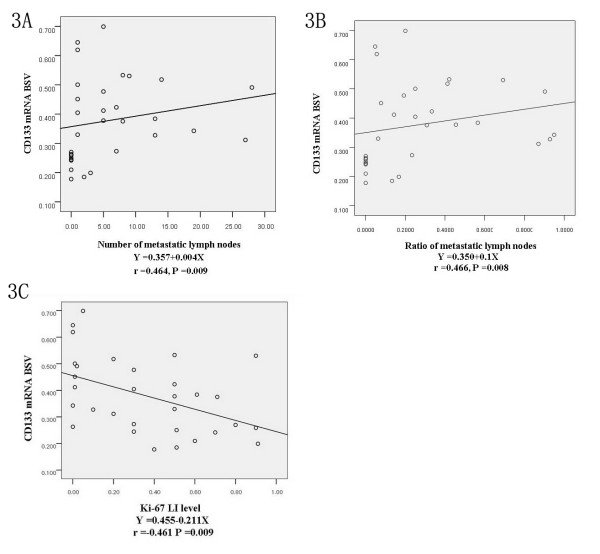
**Relation of CD133 mRNA BSV in primary lesion with lymphatic metastasis and Ki-67 LI**. Note: 3A showed relation of CD133 mRNA BSV with the number of metastatic lymph node. 3B showed relation of CD133 mRNA BSV with the ratio of metastatic lymph node. And Figure 3C showed relation of CD133 mRNA BSV with Ki-67 LI.

BSV of CD133 mRNA was significantly correlated with tumor diameter of > 5 cm (*P *= 0.041) and severer lymph node metastasis (*P *= 0.004)(Table 3). Relative analysis showed the BSV of CD133 mRNA rose with the increment of either the metastatic lymph node number (*P *= 0.009) or the metastatic lymph node ratio (*P *= 0.008) (Figure 3A and 3B).

**Table 3:** Correlation between BSV of CD133 mRNA with clinicopathological features and Ki-67 LI [n(%)] (n = 31 cases)

Parameter	Grouping	n(%)	Mean ± SD	Test value	*P *value
**Gender**	**male**	24(77.4%)	0.3674 ± 0.1292	Z = -0.520	0.603
	**female**	7(22.6%)	0.4156 ± 0.1829		
**Age(year)**	≤ **60**	10(32.3%)	0.3150 ± 0.1140	Z = -1.648	0.099
	**> 60**	21(67.7%)	0.4084 ± 0.1452		
**Tumor diameter (cm)**	≤ **5**	18(58.1%)	0.3343 ± 0.1212	Z = -2.042	0.041
	**> 5**	13(41.9%)	0.4393 ± 0.1484		
**Histological grade**	**1**	3(9.7%)	0.2555 ± 0.0095	H = 3.501	0.321
	**2**	13(41.9%)	0.3674 ± 0.1185		
	**3**	15(48.4)	0.4177 ± 0.1634		
**Invasion depth**	**T**_**1**_	1(3.2%)	0.2630 ± 0.0311	H = 3.142	0.370
	**T**_**2**_	5(16.1%)	0.3199 ± 0.1855		
	**T**_**3**_	13(41.9%)	0.4234 ± 0.1511		
	**T**_**4**_	12(38.7%)	0.3634 ± 0.1073		
**Lymph node metastasis**	**N**_**0**_	8(25.8%)	0.2395 ± 0.0309*	H = 13.583	0.004
	**N**_**1**_	12(38.7%)	0.4418 ± 0.1617		
	**N**_**2**_	7(22.6%)	0.4258 ± 0.1052		
	**N**_**3**_	4(12.9%)	0.3824 ± 0.0782		
**TNM stage**	**II**	5(16.1%)	0.3179 ± 0.1862	H = 6.409	0.093
	**II**	2(6.5%)	0.2257 ± 0.0226		
	**III**	16(51.6%)	0.3951 ± 0.1461		
	**IV**	8(25.8%)	0.4207 ± 0.0882		
**Lymphatic vessel infiltration**	**positive**	18(58.1%)	0.5013 ± 0.1412	Z = -2.142	0.040
	**negative**	13(41.9%)	0.3343 ± 0.1212		
**Vascular infiltration**	**positive**	17(54.8%)	0.4783 ± 0.1081	Z = -2.042	0.039
	**negative**	14(45.2%)	0.3343 ± 0.1212		
**Ki-67 LI**	**Lower**	16(51.6%)	0.4364 ± 0.1398	Z = -2.332	0.02
	**higher**	15(48.4%)	0.3164 ± 0.1174		

*: N_0 _*vs *N_1-3_; N_1-3 _= N_1_+N_2_+N_3 _= 0.4266 ± 0.1320

Positive staining of Ki-67 occurred in nuclei of tumor cells as sharing brown color (Figure 1G). Because average LI of Ki-67 was (36.6 ± 30.5)% in 31 patients, this value of 36.6% was applied as the bound dividing low (51.61%, 16 cases/31 cases) and high (48.39%, 15 cases/31 cases) subgroups of Ki-67 LI [14]. BSV of CD133 mRNA in low subgroup of Ki-67 LI (0.4364 ± 0.1398)% was significantly higher than that in high subgroup of Ki-67 LI (0.3164 ± 0.1174%, *P *= 0.020) (Table 3). With the increment of Ki-67 LI, BSV of CD133 mRNA gradually decreased to show the negative relation (Figure 3C).

### Prognostic analysis

**Figure 4:**
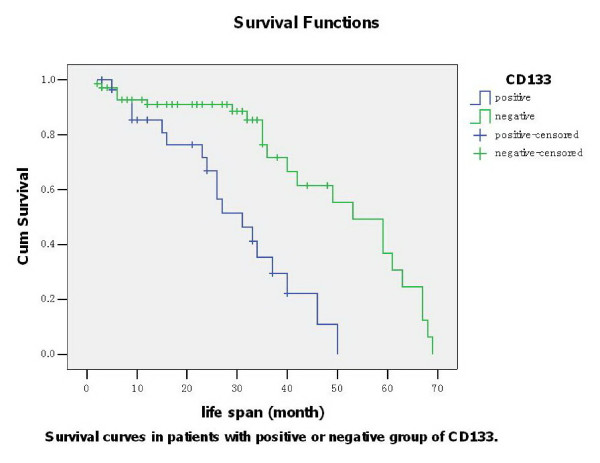
**Survival curves in different groups of CD133 protein immunostaining**. Note: *P *= 0.000 by Log rank analysis.

Univariate assessment revealed that the average survival time was (22.76 ± 13.476) months in CD133 positive subgroup while (28.41 ± 18.078) months in negative subgroup (*P *= 0.000, Figure 4). Further investigation by multivariate analysis showed that lymph node metastasis occurrence (*P *= 0.042), later stage of TNM (*P *= 0.046) and CD133 positive (*P *= 0.046) were the independent risk factors to survival respectively (Table 4).

**Table 4:** Survival analysis on CD133 protein expression and clinicopathological parameters by Cox model (n = 99 cases)

Parameter	B	SE	Wald	df	**Sig**.	Exp(B)	95.0%CI for Exp(B)
**Gender**	0.021	0.009	0.623	1	0.159	1.135	0.315~1.872
**Age(year)**	0.010	0.013	0.554	1	0.457	1.010	0.991~1.681
**Tumor diameter (cm)**	-0.076	0.070	1.186	1	0.276	0.927	0.872~1.561
**Invasion depth**	0.288	0.343	0.703	1	0.402	1.334	0.318~6.105
**Histological grade**	0.001	0.182	0.000	1	0.994	1.001	1.169~4.669
**Lymph node metastasis**	0.867	0.361	0.035	1	0.042	1.978	1.987~10.238
**TNM stage**	0.739	0.479	0.249	1	0.046	2.187	1.889~;15.312
**Lymphatic vessel infiltration**	0.871	0.592	2.168	1	0.141	2.390	0.987~6.558
**Vascular infiltration**	0.218	0.560	0.152	1	0.697	1.244	2.377~9.912
**CD133 protein expression**	0.894	0.449	3.966	1	0.046	2.445	2.118~16.381

## Discussion

CD133/prominin-1, a pentaspan transmembrane glycoprotein, has been initially described as a surface antigen specially to human hematopoietic stem cells [16] and CSCs with CD 133 positivity have been implicated in tumor progress as identified in tumor growth of pancreatic [11] and colon cancers [4]. AC133, i.e. CD133, polypeptide has a predicted size of 97 kD and contains five-transmembrane (5-TM) domains with an extracellular N-terminus and a cytoplasmic C-terminus. Whereas the expression of tetraspan (4-TM) and 7-TM molecules is well documented on mature and immature hematopoietic cells and leukocytes, this 5-TM type of structure containing two large (255-amino acid [aa] and 290-aa) extracellular loops is unique and does not share sequence homology with any known multi-TM family members [16]. Nowadays, CD133 presentation was found in many solid tumors such as brain tumor [4,7], prostate [8], pancreatic [11], hepatocellular [12] and colon cancers [5,6], but the specific role of these CSCs in tumor biology, including metastasis and recurrence, is still uncertain, especially in human GC. Although there are different phenotypes in different kinds of CSCs, the higher expression of CD133 as same phenotypes has been identified in CSCs, especially in solid tumors derived from epithelium cells of gastrointestinal organs [5-7,12,17]. O'Brien and his team [4] identified CD133 positive cells shared the characteristics of human colon cancer-initiating cells, in which CD133 positive cells were able to initiate tumor growth in minor quantity of the cells Moreover, CSCs with CD133 positivity possessed strong carcinogenesis, cloning ability and proliferating capacity as demonstrated in many experiments [4-8,11,12,17], and were resistant to anti-cancer therapy [10,18]. Hence, the metastasis and recurrence of cancer as one of main factors inflecting on the prognosis has still been hard to be overcome thoroughly until now.

Regulation and control on gene expression is partially dependent on the transcript and the protein expression appearances. As reported [6], the initiation and the proliferation of colorectal cancer were based on CSCs with CD133 positive only in minor quantity, which was also identified not only in prostate [8], pancreatic [11] and hepatocellular [12] cancers but also in gastric cancer [12,19]. In this study of ours, CD133 protein positive structures had been seen in 29.3% cases in primary lesion of 99 patients' group, but no CD133 positive structures in NCGT. Simultaneously, CD133 mRNA expression had been identified in all primary lesions of 31 patients' group, but only 16.1% cases in NCGT of this same group. As compared with the level of CD133 mRNA BSV in NCGT, this value was significantly higher in primary lesion. Additionally, CD133 expression significantly correlated with tumor diameter of > 5 cm, later TNM stage and T_3_-T_4 _as stratified analysis. Furthermore, either severer invasion depth or later TNM stage was the independent risk factor for CD133 protein expression. Therefore, it can be concluded from the above mentioned results that the tumor cells with CD133 protein and CD133 mRNA may play some important roles in the growth and the invasion of GC in human being.

Hermann PC et al [11] demonstrated that a subpopulation of migrating CSCs with both CD133 positive and CXCR4 positive was essential for tumor metastasis of pancreatic adenocarcinoma. Mehra N et al [20] examined whether RNA expressions of CD133 and CD146, a pan-endothelial marker, were increased in the blood of cancer patients and whether these factors correlated with patient characteristics and were predictive factors of survival. Their results in the peripheral blood mononuclear cells of 131 progressive cancer patients, 37 healthy volunteers, and 5 patients who received granulocyte colony-stimulating factor showed that patients with metastatic disease had a significant increase in CD133 mRNA (*P *= 0.03), specifically patients with bone metastasis (*P *< 0.001). In a recent study, it had been examined whether increased levels of expression of CD133 mRNA by semi-quantitative real-time RT-PCR analysis in peripheral blood predicted disease recurrence in patients with colon cancer. Their results indicated that elevated CD133 mRNA levels predicted colon cancer recurrence as an independent factor in Stage IV of TNM disease [21]. Similarly, the higher level of CD133 mRNA in primary lesion occurred in subgroup with lymph node metastasis, and this elevated level was positively relevant to the increments of metastatic lymph node ratio or metastatic lymph node number as demonstrated in our results of this study. Additionally, CD133 positive cells in cancerous emboli in vessel-like structures had been observed morphologically as a first report in our knowledge. In the immunohistochemical investigation in this study, CD133 positive percentage in subgroup of lymph node metastasis was significantly higher than that in subgroup without lymph node metastasis. With the increments of tumor invasion depth and TNM stage, CD133 protein positivity rate increased. From such results, it can be concluded that the positive expressions of CD133 mRNA and CD133 protein positively related to the lymphatic metastasis in GC, which can reasonably be considered as a risk factor to lymphatic metastasis and tumor invasion. Hence, the strategies aimed at the CD133 and SDF-1/CXCR4 modulating axis, and the molecular pathway for lymphatic metastasis may have important clinical significances to inhibit metastasis of CSCs.

Ki-67 is a kind of nuclear protein, which expresses in cellular cycle of G_1_, S, G_2 _and M phases, but not in G_0 _phase. In order to probe the relation of CD133 expression with the proliferation of tumor cells with or without CD133 positivity, the CD133 mRNA expressive level was applied in this study due to the rare CSCs (usually around 1%-5% of total tumor cells) with CD133 protein positivity in tumor as common and the difficulty to identify CSCs as immature tumor cells from matured tumor cells morphologically. From current limited information indicated in this investigation of ours, there occurred the significantly higher expression of CD133 mRNA in subgroup with lower Ki-67 LI in comparison with that in subgroup with higher Ki-67 LI. Theoretically, this phenomenon observed in our study could be elucidated as the various biological profiles in different stage of tumor differential process or in proliferating characterization in the early stage of carcinogenesis and tumor development. And this proliferating characterization would be gradually weakened in tumor development probably. Additionally, in some extent, this higher expression of CD133 mRNA in subgroup with lower Ki-67 LI could also be explained to the resistant potential of CSCs to anti-cancerous therapy because tumor cells in Phase G_0 _such as most of CSCs were difficult to be killed by cytotoxin drugs and radiotherapy [18]. On the other hand, for other explanation of this interesting phenomenon with negative relation between CD133 mRNA and Ki-67 LI, as our consideration, it is also contributed to the different proliferating abilities of matured tumor cells and immature tumor cells of CD133 positivity with some characteristics of CSCs. As well known, CSCs possessed strong differentiation proficiency, but this proficiency might not mean strong proliferating ability, especially comparing with that of matured tumor cells with CD133 negative expression probably. As there occurred so many kinds of cells in primary lesion and the limitation of only morphological and immunohistochemical observations in this study, the investigation on the both expressions of CD133 and Ki-67 in the same tumor cells should be necessarily considered to carry out in future.

As reported, cancer patients with high CD133 mRNA expression, using a defined cutoff value, showed a decreased survival compared with patients with low or undetectable CD133 expression (21% versus 45% cumulative survival, respectively, after 20 months; *P *= 0.01). Among patients with metastasis to the bone, cumulative survival was only 22%, compared with 61% for patients with low or undetectable CD133 levels (*P *= 0.004) [20]. Furthermore, multivariate analysis in their study showed that CD133 expression was an independent predictor for overall survival in patients with bone metastases [20]. At the same time, they compared the level of CD146 mRNA, a pan-endothelial marker, with the level of CD133. CD146 mRNA level was not increased in patients with cancer, nor did CD146 mRNA level correlate with clinical variables or survival [20]. In this study of ours, prognostic analysis based on the different subgroups with or without CD133 protein positivity was assessed by univariate and multivariate evaluations. Univariate assessment revealed that average survival time was (22.76 ± 13.476) months in CD133 positive subgroup while (28.41 ± 18.078) months in negative subgroup. Multivariate analysis showed that, excepting for lymph node metastasis occurrence and later stage of TNM, CD133 protein positivity was also an independent risk factor to survival. Obviously, the detection of CD133 tumor marker regarding as one of the markers of CSCs may be a useful and supplementary means to take a judgment to prognosis of GC.

## Conclusion

The expressions of CD133 protein and CD133 mRNA correlated with severer lymph node metastasis and lower LI of Ki-67. Positive expression of CD133 protein indicated the poorer prognosis, which raised the possibility that CD133 positive cells might execute some functions to promote the lymphatic metastasis in patients with GC. However, the study about the CSCs, especially the tumor cells with CD133 positivity, is still in the initial stage in GC, and the biological profiles of CSCs of gastric cancer should be further investigated in novel diagnosis, more suitable treatment strategies including the application of gene therapy by CD133 target and prognostic judgment in order to improve the effect of treatment on gastric cancer.

## Competing interests

The authors declare that they have no competing interests.

## Authors' contributions

PZ contributed in study design, definition of intellectual content, literature research, experimental studies, data acquisition, data analysis, statistical analysis and manuscript preparation. JGW and SHW contributed in literature research, study design and data analysis. PZ, JGW, XQL contributed in pathological and immunohistochemical observations. PZ, JGW, RQL contributed in RT-PCR analysis. STW contributed in technique supports in laboratory. XCN, JWY, and BJJ contributed in clinical managements. BJJ and JWY contributed in grants for this study, guarantor of integrity of the entire study, study concepts, study design and manuscript review. All authors read and approved the final manuscript for publication.
